# Hypnotic Effect of *A*. *absinthium* Hydroalcoholic Extract in Pentobarbital-Treated Mice

**DOI:** 10.1155/2021/5521019

**Published:** 2021-04-21

**Authors:** Hassan Rakhshandeh, Amirhossein Heidari, Ali Mohammad Pourbagher-Shahri, Roghayeh Rashidi, Fatemeh Forouzanfar

**Affiliations:** ^1^Pharmacological Research Center of Medicinal Plants, Mashhad University of Medical Sciences, Mashhad, Iran; ^2^Medical Toxicology Research Center, Faculty of Medicine, Mashhad University of Medical Sciences, Mashhad, Iran; ^3^Neuroscience Research Center, Mashhad University of Medical Sciences, Mashhad, Iran; ^4^Department of Neuroscience, Faculty of Medicine, Mashhad University of Medical Sciences, Mashhad, Iran

## Abstract

**Background:**

Current drugs used in the management of insomnia are associated with side effects. The use of medicinal herbs for insomnia treatment has recently been suggested.

**Objective:**

The present study aimed to determine the hypnotic activity of the hydroalcoholic extract of *Artemisia absinthium (A*. *absinthium)* in mice.

**Method:**

The toxicity of *A*. *absinthium* extract is assessed by their lethal dose 50% (LD50), and cytotoxicity evaluation was also done with PC12 cell lines by MTT assay. *A*. *absinthium* extract (25, 50, 100, and 200 mg/kg) and 3 fractions (*n*-butanol fraction (NBF), ethyl acetate fraction (EAF), and aqueous fraction (AQF)) were administered intraperitoneally30 minutes before 30 mg/kg pentobarbital intraperitoneal injection; after that, the sleeping time and sleep latency were recorded.

**Results:**

The LD50 value was 2.4 g/kg. The extracts tested showed no negative effect on the proliferation of PC12 cells. *A*. *absinthium* extract increased the duration of pentobarbital-induced sleep at doses of 100 and 200 mg/kg (*P* < 0.01-*P* < 0.001). Similarly, AQF, EAF, and NBF at 200 mg/kg could increase sleep duration (*P* < 0.05). The sleep latency was decreased by *A*. *absinthium* extract at doses of 100 and 200 mg/kg (*P* < 0.05-*P* < 0.01), AQF (*P* < 0.05), and EAF (*P* < 0.05). Besides, flumazenil reversed the hypnotic effect of *A*. *absinthium* extract (*P* < 0.05).

**Conclusion:**

*A*. *absinthium* extract probably demonstrated sleep-enhancing effects by regulating GABAergic system.

## 1. Introduction

Insomnia has a negative impact on the quality of life leading to impairment in memory, mood disorders, and longer reaction [1–3]. Controlled-release melatonin, doxepin, z-drugs (zolpidem, eszopiclone, and zaleplon), and the orexin receptor antagonist suvorexant are major drug classes used to treat insomnia. Although these hypnotic drugs may improve sleep quality, they are associated with unwanted side effects [4]. The use of herbal products, as one element of complementary and alternative medicine, is increasing globally [5]. *Artemisia absinthium* (*A. absinthium*) (Wormwood) is an important perennial shrubby medicinal plant that is globally distributed in Europe, Asia, Middle East, and North Africa [6]. *A. absinthium* is named by numerous common names. It is named as wormwood, green ginger, absinthe, absinthium, in English; Genepi in Latin; Absinth, Wermut in German; Damseeh and Afsanteen in Arabic [6]. *A. absinthium* is employed in folk medicine as a febrifuge, antiseptic, antispasmodic, anthelmintic, cardiac stimulant, for repairing deteriorating mental function and memory improvement [7, 8]. Recently, it has shown various pharmacological effects including neuroprotective [9], antidiabetic [10], and hepatoprotective [11] properties. Other *Artemisia* species such as *A. persica* showed antianxiety [8] effects. *A. absinthium* contains compounds such as terpenoids (e.g., linalool), flavonoids (e.g., quercetrin) that these constituents have been reported to have hypnotic effects [12].The barbiturate pentobarbital binds to gamma-aminobutyric acid type A (GABA(A)) receptors to potentiate the effects of GABA [13]. Pentobarbital-induced hypnosis in mice is a popular animal model for screening of compounds with suspected sedation/hypnotic properties [14]. Therefore, the current study was intended to estimate the sleep-prolonging effect of *A. absinthium* and its fractions. Furthermore, the possible neurotoxicity of the plant was evaluated using a rat pheochromocytoma PC12 cell line.

## 2. Material and Methods

### 2.1. Reagents and Chemicals

Pentobarbital sodium, flumazenil, 3-(4,5-dimethyl thiazole-2yl)-2,5-diphenyl tetrazolium bromide (MTT), and penicillin-streptomycin were bought from Sigma (USA). Dulbecco's Modified Eagles Medium (DMEM), fetal bovine serum (FBS), and other cell culture material were bought from Gibco Life Technologies (USA). Diazepam was obtained from Chemidarou Company (Iran). Tween 80 was purchased from Merck (Germany).

### 2.2. Plant and Extract

Aerial parts of *A. absinthium* were collected from the Allah Akbar mountain (Khorasan Razavi Province of Iran). The plant was identified in the Herbarium of Khorasan Razavi Agricultural and Natural Resources Research Center. A voucher specimen (No. 11856) has been deposited in this institute. The powder of the aerial parts of *A*. *absinthium* was percolated with ethanol 70% for 72 hr. After extraction, the solvents were removed [15]. A part of dried *A*. *absinthium* was further subjected to solvent-solvent extraction for three fractions, namely, *n*-butanol fraction (NBF), ethyl acetate fraction (EAF), and aqueous fraction (AQF). The yield of AQF, NBF, and EAF was 55%, 18%, and 27%, respectively. *A*. *absinthium* fractions were dried on a water bath. The *A*. *absinthium* extract and its fractions were dissolved in normal saline comprising 1% Tween 80 [16].

### 2.3. Animals

Male albino mice weighing 25–35 g were maintained at 22 ± 1°C with 12 hr of light and 12 hr of dark and had free access to water and food. Experiments were carried out in accordance with the ethical guidelines approved by the Animal Care Use Committee of Mashhad University of Medical Sciences (981134).

### 2.4. Grouping and Treatment

A total of 66 adult male albino mice were divided into 6 groups as below:

  Group 1: Control (normal saline)
  Group 2: Positive control or Diazepam (3 mg/kg)
  Groups 3–6: Treatment group or different doses of *A. absinthium* extract (25, 50, 100, and 200 mg/kg)
  Groups 7–9: Treatment group with AQF, EAF, and NBF (200 mg/kg)
  Group 10: Diazepam (3 mg/kg) plus flumazenil (2 mg/kg)
  Group 11: *A. absinthium* extract at a dose of 200 mg/kg plus flumazenil (2 mg/kg)

### 2.5. All Agents Were Administrated Intraperitoneally

Thirty min after the intraperitoneal administration of the agents, pentobarbital (30 mg/kg IP) was administrated to induce sleep. Time elapsed between the administrations of pentobarbital until loss of righting reflex and immobility was considered as the sleep latency, while the time from the loss to its recovery was considered the sleeping time. Mice were given flumazenil (2 mg/kg) 30 min before diazepam or *A*. *absinthium* extract administration [17].

### 2.6. Toxicity Assessment

The possible neurotoxicity of *A*. *absinthium* extract and its fractions was tested on PC12 cells. Cells were seeded in 96-well plates in DMEM culture medium supplemented with 10% FBS and 1% penicillin streptomycin. After 24 hours, the culture medium was changed to fresh one having vehicle (1% Tween 80) or *A*. *absinthium* extract (200, 400, and 800 *μ*g/mL) and its fractions (800 *μ*g/mL). After 24 hours, the MTT (0.5 mg/mL in final volume) was added to culture medium of each well and incubated for 2 hours. Afterwards, the medium was discarded and DMSO was added to each well to dissolve any formazan crystals formed. The absorption of dye was measured at 570 nm [15]. For the determination of LD50, a series of doses of *A*. *absinthium* extract were administered intraperitoneally to mice. The number of deaths in each group within 24 hours was recorded. The maximum dose which did not cause death and the minimum dose causing death of one animal were noted. The average of these two doses was defined as the median lethal dose [18].

### 2.7. Data Analysis

All results are presented as mean ± SEM. Statistical analysis was done using one-way analysis of variance (ANOVA) followed by Tamhane's T2 post-hoc test. Values with *p* < 0.05 were considered as statistically significant.

## 3. Results

### 3.1. Assessment of Toxicity

The highest dose of *A*. *absinthium* extract that did not result in death in any mouse and the lowest dose that resulted in death in one mouse were 1.6 and 3.2 g/kg, respectively. The LD50 value of *A*. *absinthium* extract was 2.4 g/kg. Results showed no significant decrease in the proliferation of PC12 cells at the concentrations of 100, 200, 400, and 800 *μ*g/ml of the extract and 800 *μ*g/ml of the fractions (Table 1).

### 3.2. Effects of *A*. *absinthium* Extract on Pentobarbital-Induced Sleeping Behaviors in Mice

The results show that diazepam (3 mg/kg) and *A*. *absinthium* extract (100 and 200 mg/kg) significantly increased the sleep time compared with the control group (*P* < 0.001, *P* < 0.01, and *P* < 0.001, resp.). Flumazenil (2 mg/kg) and *A*. *absinthium* extract (200 mg/kg) significantly reversed the effects of the diazepam (3 mg/kg) (*P* < 0.001) (Table 2).

As illustrated in Table 3, diazepam (3 mg/kg) and *A*. *absinthium* extract (100 and 200 mg/kg) significantly reduced the sleep latency compared with the control group (*P* < 0.001, *P* < 0.05, and *P* < 0.01, resp.). Flumazenil (2 mg/kg) and *A*. *absinthium* extract (200 mg/kg) significantly reversed the augmented effects of diazepam (3 mg/kg) in pentobarbital-treated mice (*P* < 0.001 and *P* < 0.05, respectively) (Table 3).

Effects of *A. absinthium* extract fractions on pentobarbital-induced sleeping behaviors in mice are as follows:

The results show that all fractions (200 mg/kg) significantly increased the sleep time compared with the control group (*P* < 0.05) (Table 4). EAF and AQF (200 mg/kg) significantly reduced the sleep latency compared with the control group (*P* < 0.05) (Table 5).

## 4. Discussion

Based on experimental result, *A*. *absinthium* extract reduced the sleep latency and augmented the total sleep in pentobarbital-induced sleeping mice. Previous studies have shown that a wide range of herbal medicine ingredients including alkaloids, flavonoids (e.g., quercetrin and luteolin), terpenoids (e.g., linalool), and steroids have hypnotic properties [12]. *A. absinthium* contains numerous phytochemical ingredients, namely, lactones, terpenoids (e.g., *γ*-terpinene, 1,4-terpeniol, *trans*-thujone, cadinene camphene, bornyl acetate, *trans*-sabinyl acetate, myrcene, guaiazulene, chamazulene, camphor, and linalool), essential oils, tannins, organic acids, resins, and phenols. The plant also have flavonoids (e.g., quercetin), flavonoid glycosides such as quercitin-3-O-D-glucoside, isorhamnetin-3-O-rhamnose glucoside, quercetin-3-O-rhamnoglucoside, isoquercitrin, and isorhamnetin-3-O-glucoside, and phenolic acids (coumaric, chlorogenic, syringic, salicylic, and vanillic acids) [6, 19, 20]. Benzodiazepines such as diazepam enhance the effect of the neurotransmitter gamma-aminobutyric acid (GABA) at the GABAA receptor and thereby increase pentobarbital-induced sleeping time [21]. Indeed, GABA opens the Cl− channels accompanied by a membrane hyperpolarization that cause CNS depression and sedative and hypnotic action [22, 23]. Therefore, drugs that affect these systems can be considered in the treatment of insomnia. Accordingly, we observed that pretreatment of mice with flumazenil (as the antagonist of GABAA benzodiazepine receptor) reduces the prolonged sleep effect of diazepam. Furthermore, we found that flumazenil reverses the hypnotic effect of *A. absinthium* extract. We prepared three fractions from *A. absinthium* extract: the AQF, which contains solubilizing the polar agents and water-soluble plant ingredients (e.g., glycosides, tannins, and quaternary alkaloids); EAF, which contains ingredients with intermediate polarity; and the NBF, which contains nonpolar agents like sterols, alkanes, and some terpenoids [14]. In our study, all fractions augmented sleep duration, suggesting that the extract and its various fractions may have bioactive constituents with hypnotic property. To evaluate the fact that the hypnotic effect is associated with no negative impact on neurons, we utilized PC12 cells. No cytotoxic effect for *A*. *absinthium* extract was found at 24 h period in PC12 cells. Acute toxic study demonstrated the lethal dose (LD (50)) of *A*. *absinthium* extract was 2.4 g/kg. Based on these results, *A. absinthium* possesses hypnotic activities in a relatively safe dose range.

## 5. Conclusion

Taken together, all the data presented here demonstrate that *A*. *absinthium* extract increased hypnotic effects in pentobarbital-treated mice. The GABAergic system might be involved in the mechanisms of these effects. Further studies are needed to support this evidence.

## Figures and Tables

**Table 1:** Effects of *A. absinthium* on viability of PC-12 cell. Values are expressed as the mean ± SEM (*n* = 4).

Saline	Extract 200	Extract 400	Extract 800	EAF 800	NBF 800	AQF 800
100.0 ± 2.198	93.75 ± 1.315	93.25 ± 1.797	95.00 ± 1.080	94.00 ± 1.472	96.00 ± 0.7071	93.25 ± 1.377

**Table 2:** Effects of *A. absinthium* extract (25, 50, 100, and 200 mg/kg) or diazepam (3 mg/kg) on duration of sleep. Values are expressed as the mean + SEM (*n* = 6). ^*∗∗*^*p* < 0.01 and ^*∗∗∗*^*p* < 0.001 vs. saline; ^###^*p* < 0.001 vs. the same group without flumazenil (2 mg/Kg).

Saline	Diazepam	Diazepam + flumazenil	Extract 25	Extract 50	Extract 100	Extract 200	Extract 200+ flumazenil
21.33 ± 1.909	40.33 ± 1.022 *∗∗∗*	22.00 ± 0.6831###	22.83 ± 0.9458	25.67 ± 1.687	30.67 ± 2.275 *∗∗*	32.50 ± 1.176 *∗∗∗*	22.67 ± 1.453 ###

**Table 3:** Effects of *A. absinthium* extract (25, 50, 100, and 200 mg/kg) or diazepam (3 mg/kg) on the sleep latency. Values are expressed as the mean + SEM (*n* = 6). ^*∗*^*p* < 0.05, ^*∗∗*^*p* < 0.01, ^*∗∗∗*^*p* < 0.001 vs. saline; ^#^*p* < 0.05, ^###^*p* < 0.001 vs. the same group without flumazenil (2 mg/kg).

Saline	Diazepam	Diazepam + flumazenil	Extract 25	Extract 50	Extract 100	Extract 200	Extract 200 + flumazenil
8.833 ± 0.4773	3.667 ± 0.2108 *∗∗∗*	8.333 ± 0.5578 ###	8.667 ± 0.5578	7.833 ± 0.4773	5.833 ± 0.8333 *∗*	5.333 ± 0.7149 *∗∗*	8.5 ± 0.6#

**Table 4:** Effects of aqueous fraction (AQF), ethyl acetate fraction (EAF), or n-butanol fraction (NBF) of *A. absinthium* extract on the duration of sleep. Values are expressed as the mean + SEM (*n* = 6). ^*∗*^*p* < 0.05 vs. saline.

Saline	EAF	NBF	AQF
21.33 ± 1.909	28 ± 1 ^*∗*^	28.33 ± 1.16 ^*∗*^	28 ± 1.4^*∗*^

**Table 5:** Effects of aqueous fraction (AQF), ethyl acetate fraction (EAF), or n-butanol fraction (NBF) of *A. absinthium* extract on sleep latency. Values are expressed as the mean + SEM (*n* = 6). ^*∗*^*p* < 0.05 vs. saline.

Saline	EAF	NBF	AQF
8.833 ± 0.4773	5.8 ± 0.9 ^*∗*^	6.5 ± 0.67	5.8 ± 0.7 ^*∗*^

## Data Availability

Some data will be available from the corresponding author upon reasonable request.
